# Time Dependent Assessment of Morphological Changes: Leukodepleted Packed Red Blood Cells Stored in SAGM

**DOI:** 10.1155/2016/4529434

**Published:** 2016-01-21

**Authors:** Ibrahim Mustafa, Asma Al Marwani, Khuloud Mamdouh Nasr, Noora Abdulla Kano, Tameem Hadwan

**Affiliations:** ^1^Health Sciences Department, College of Arts and Sciences, Qatar University, Doha, Qatar; ^2^Hematology Department, Hamad General Hospital, Hamad Medical Corporation, Doha, Qatar

## Abstract

Usually packed red blood cells (pRBCs) require specific conditions in storage procedures to ensure the maximum shelf life of up to 42 days in 2–6°C. However, molecular and biochemical consequences can affect the stored blood cells; these changes are collectively labeled as storage lesions. In this study, the effect of prolonged storage was assessed through investigating morphological changes and evaluating oxidative stress. Samples from leukodepleted pRBC in SAGM stored at 4°C for 42 days were withdrawn aseptically on day 0, day 14, day 28, and day 42. Morphological changes were observed using scanning electron microscopy and correlated with osmotic fragility and hematocrit. Oxidative injury was studied through assessing MDA level as a marker for lipid peroxidation. Osmotic fragility test showed that extended storage time caused increase in the osmotic fragility. The hematocrit increased by 6.6% from day 0 to day 42. The last 2 weeks show alteration in the morphology with the appearance of echinocytes and spherocytes. Storage lesions and morphological alterations appeared to affect RBCs during the storage period. Further studies should be performed to develop strategies that will aid in the improvement of stored pRBC quality and efficacy.

## 1. Introduction

Storage of red blood cells in preservative medium is associated with harmful metabolic, biochemical, and molecular changes to erythrocytes: these changes are collectively referred to as “storage lesions” [1, 2]. Blood products such as RBC stored with additive solutions in different temperatures contribute storage lesions significantly [3]. The most probable sites of damage will be cytoskeletal proteins in RBC membrane [4]. These membrane changes will lead RBC to be fragile and increase osmotic fragility and changes in electrolyte imbalance. Great efforts have been done to provide a suitable and a safe supply of blood with more benefits than side effects. Currently, the storage procedures of blood bags in blood banks require some conditions to ensure the maximum storage time for a healthy and safe blood supply. Nowadays, blood bags can be stored up to 42 days at 2–6°C, as long as the mean hemolysis does not exceed 0.8% and more than 75% red blood cells survive in the first 24 hours after transfusion [5] However, pathological consequences can affect the stored blood; they are termed as storage lesions. Storage lesions are hypothesized to decrease the efficiency of stored blood and decrease their ability to act their required role after transfusion, but these hypotheses have no clear evidences yet [6]. Some reversible changes result from stored blood such as decreased ATP and 2,3-DPG. However, some other damage is irreversible and includes increased osmotic fragility, small echinocytic rigid red blood cells with reduced function, microvesiculation, and hemolysis. Leukoreduced packed red blood cells will have storage lesions due to lipid peroxidation of RBC membrane and that will result in morphological alterations in stored blood [7]. Storage lesions in stored red blood cells are now considered well-known phenomena, yet there are structural and functional aspects of stored red blood cells to be explained. In this study, we look primarily to see the progressive irreversible changes in RBC morphology to correlate specially RBC SEM pictures hemolytic lytic lesions we observed to osmotic fragility changes during the course of storage time. Also we want to correlate the observed changes in hematocrit to morphological alterations.

## 2. Materials and Methods

### 2.1. Blood Sample Collection

The blood bags were obtained from Hamad Medical Cooperation (HMC) blood donor centre at Doha, Qatar. Institutional review board (IRB) approval was obtained from HMC for using the donor's blood for the purpose of research. Whole blood, 450 mL, was collected from healthy volunteer donors in citrate phosphate dextrose adenine (CPDA-1) anticoagulant, according to standard blood bank procedures. The units included in the study were leukodepleted red blood cells concentrate suspended in 100 mL SAGM with a final volume of ~300 mL of packed red blood cells (pRBCs). RBC units were stored at 4–6°C for 42 days and samples were withdrawn aseptically on day 0, day 14, day 28, and day 42 for the investigations.

### 2.2. Determination of Hematocrit (HCT)

Well-mixed pRBC was drawn into two microhematocrit capillary tubes by capillary action avoiding air bubbles. Three-fourths of the tubes were filled and excess blood was wiped with a gauze. One end of each tube was sealed with a small amount of clay material at a 90° angle, ensuring that the seal had a perfectly flat bottom. The filled and sealed capillary tubes were placed into the hematocrit centrifuge, the sealed ends pointing toward the outside of the centrifuge. Duplicate samples were placed opposite each other in order to balance the centrifuge and the position number of each specimen was recorded. Capillary tubes were centrifuged at 10,000 rpm for 5 minutes at room temperature, using Haematokrit 210 centrifuge. Hematocrit was measured directly from the tubes using the hematocrit reading scales.

### 2.3. Osmotic Fragility Test

RBC osmotic fragility was determined by the method of established osmotic fragility assay; briefly pRBC is added to 14 tubes containing saline (NaCl) with increasing concentration from 0–0.9% at pH 7.4. The tubes were well mixed and incubated at room temperature for 1 hour. The tubes were then centrifuged at 3,000 rpm for 5 min, and supernatants were collected. Optical density of the supernatant was measured by spectrophotometer at 540 nm. Hemolysis in each tube was expressed as a percentage, taking 100% the maximum value of absorbance of the distilled water (0% concentration). The erythrocytes treated with normal saline were used as a negative control (0% hemolysis), and those treated with distilled water were used as a positive control (100% hemolysis).

### 2.4. Scanning Electron Microscopy (SEM)

Blood samples from the blood bags were prepared for SEM as per the established protocols. The preparations are basically fixation and dehydration of the samples. In the fixation, blood samples from each blood bag were exposed to the fixative agent 3% glutaraldehyde with incubation in 4°C for 60 minutes. Then, the samples were centrifuged at low speed to remove the glutaraldehyde supernatant. The glutaraldehyde needs to be totally removed, so the samples were washed 3 times with saline to remove the remnant of fixative agent. After fixation, dehydration takes place. The samples were dehydrated with different concentrations of ethanol, starting from 50% to 100%. After adding each concentration, the samples were incubated at 4°C for 15 minutes and then centrifuged to remove the ethanol supernatant. The 100% ethanol step was done 3 times, and the incubation time was 30 minutes to ensure total dehydration. The blood samples were kept in the last 100% ethanol and at the central laboratory in Qatar University for the scanning electron microscopy was done to see the morphological changes in red cells.

### 2.5. RBC Membrane Ghost Preparation

This method is also known as Dodge membrane preparation. It yields RBC membranes concentrates in white color without hemoglobin. This method is used to study the proteins and lipids in the RBC membrane. The ghost membranes were obtained by Dodge, Mitchell, and Hanahan's method. Blood samples were first centrifuged to separate remnant plasma and then washed 3 times with saline at 3000 rpm for 5 minutes. A specific volume of washed red blood cells was added to Eppendorf tubes and suspended in ice-cold 5 mM phosphate buffer of pH 8. The tubes were then incubated for 10 minutes in ice bucket. After incubation, they were centrifuged in Eppendorf centrifuge (5424 R) at speed 15000 g at 4°C for 10 minutes. Then, the hemolysate supernatant is discarded, keeping the pallet that contains RBC membranes. The steps of washing were repeated around 5 times until the color of the membranes becomes very pale yellow to white. After the last wash, membrane concentrates were kept in the Eppendorf tubes and stored in −80°C. The prepared ghost RBCs were used in the TBARS assay.

### 2.6. Determination of Thiobarbituric Acid Reactive Substances (TBARS)

One of the consequences of an oxidative stress process is an increase in lipid peroxidation. TBARS stands for thiobarbituric acid reactive substances, which are by-products resulting from lipid peroxidation caused by oxidative stress. In this respect, lipid peroxidation is caused by an attack of free radicals upon cell membrane lipids. One of the substances commonly measured as a marker for oxidative stress is malondialdehyde (MDA). Measurement of MDA, the most abundant product arising from lipid peroxidation, has been extensively used as an index of oxidative stress. In this method, the lipid peroxidation MDA assay kit from Sigma-Aldrich was used. The ghost membranes samples were thawed at room temperature, and different concentrations of MDA standards from the kit were prepared to help finding the concentration of MDA from the samples. MDA lysis buffer was added on a specific volume of the samples to release MDA present. To remove the turbidity cause by the proteins, perchloric acid 2 N was used to precipitate them. They were centrifuged at 13,000 g for 10 minutes and supernatants were aspirated to new tubes. TBA solution was added to the samples and the standards to form the TBA-MDA adduct and then incubated for 60 minutes at 95°C using the hot plate. After that, the samples reactions and standards were pipetted to 96-well plates for reading. Using TECAN plate reader, the absorbance was measured at 532 nm.

### 2.7. Statistical Analysis

The software SPSS (IBM SPSS Statistics, version 22) was used to compare between the mean results of each day applying one-way ANOVA. Most of the methods conducted were tested for triplicate aliquots from each blood bag. The results are expressed as the mean (per day) ± SEM.

## 3. Results and Discussions

### 3.1. Structural Changes

**Figure 1:**
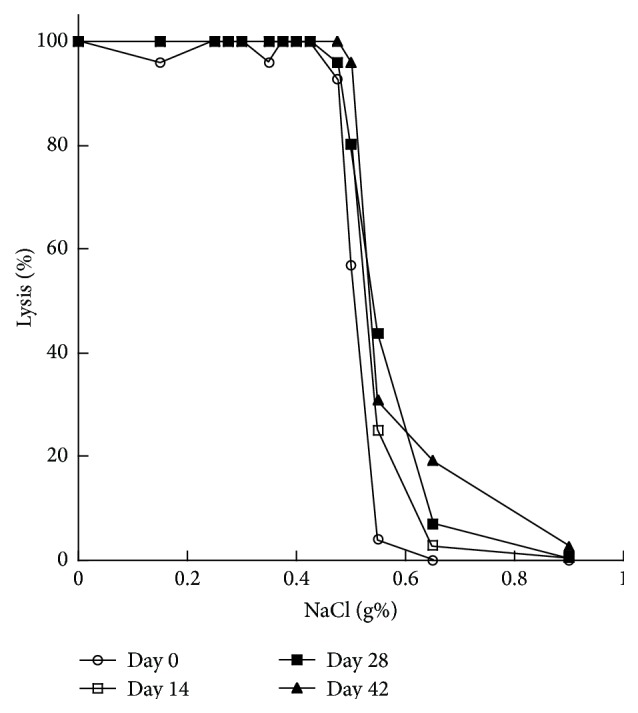
Erythrocytes osmotic fragility for leukoreduced packed RBC on standard method. The graph represents the measurements of osmotic fragility of stored pRBCs of different days. Day 0 normal range is indicated by the line with circle representing the lowest values of osmotic fragility, while the consecutive days show gradual shifting to the right, indicating an increase in osmotic fragility.

**Figure 2:**
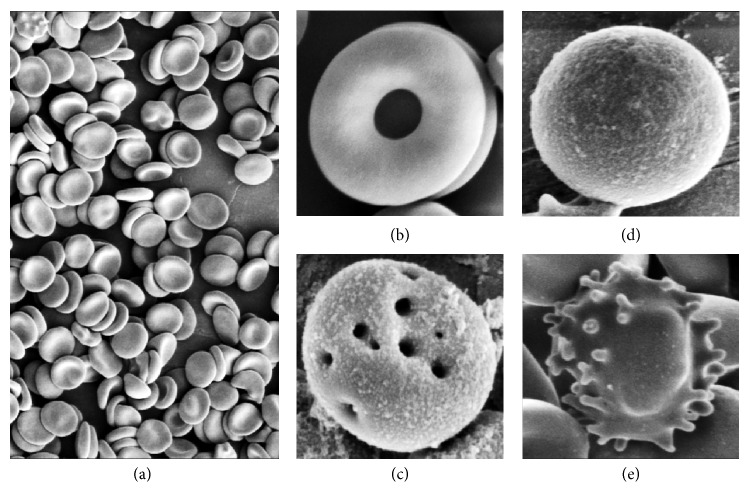
Scanning electron micrographs of erythrocytes with signs of hemolysis. Micrograph (a) shows RBCs with normal biconcave shape without any sign of damage or lesions on day 0 on low magnification 2500x. However, the cells in micrographs (b and c) demonstrate a sign of hemolysis, punctate (tiny) holes in the membrane of RBCs. The micrographs (d and e) represent spherocytosis and echinocytes, respectively. The micrographs from (b) to (e) are on day 28 and on higher magnification.

Investigating storage lesions and oxidative stress on RBCs is essential to assess the extent of damage to the cells, as this damage causes alterations and decreases the functionality and viability of the red blood cells after transfusion. The principle of osmotic fragility is to measure the resistance of RBCs to increasing osmotic stress. As shown (Figure 1), the leftmost curve represents the measurements of fresh blood of day 0 with the most resistance to increasing dilutions of NaCl. Osmotic fragility increases with prolonged storing of the blood bag, which is depicted in the figure. Days 14, 28, and 42 show shifting towards the right, indicating increased fragility, with day 42 having the least resistance to hypotonicity (the rightmost curve) and earliest hemolysis. The osmotic fragility test is used to determine the susceptibility of red blood cells to osmotic stress. The shape of RBC mainly influences the resistance of RBC to hemolysis. In case of old red blood cells stored for 42 days, they lose the normal morphology of discoid shape and progress to spherocytes, with decreased surface area to volume ratio. Spherocytes can hemolyze in hypotonic environment earlier than normal shaped RBC. The main defect that occurs in old RBCs is in the proteins that connect the cytoskeleton to the membrane, which are spectrin, ankyrin, protein 4.2, and band 3 [8]. The increased production of reactive oxygen species with decreased antioxidants causes the oxidation and degradation of these proteins. This damage can result in losing parts from the unsupported membrane in the form of microvesicles, leading to decrease in surface area to volume ratio. In consequence, spherocytes will have high osmotic fragility with increased hemolysis (Figure 2). Biochemical changes such as reduced ATP concentration and increase in calcium can contribute to increase rigidity of the RBCs. In the body, these cells will have decreased viability because they are inflexible and unable to pass through the small blood vessels. In this study, a progressive increase in the osmotic fragility was observed in day 42; it showed the lowest osmotic resistance with shifting the curve towards the right, while fresh RBCs have lower osmotic fragility that can be evidenced by seeing the curve on the left. This means that older red blood cell will be more fragile and will lyse on slightly decreased sodium chloride concentration that fresh RBCs can tolerate. These results are in agreement with a study published by Blasi et al. [7]. In their study, they tested leukoreduced packed RBC to evaluate the changes in RBC morphology during storage. They performed osmotic fragility test. They obtained the same result, showing that prolonged storage produces increase in the osmotic fragility and causes the curve to shift to the right.

### 3.2. Changes in Packed Cell Volume and Scanning Electron Microscopy: Morphological Alterations

**Figure 3:**
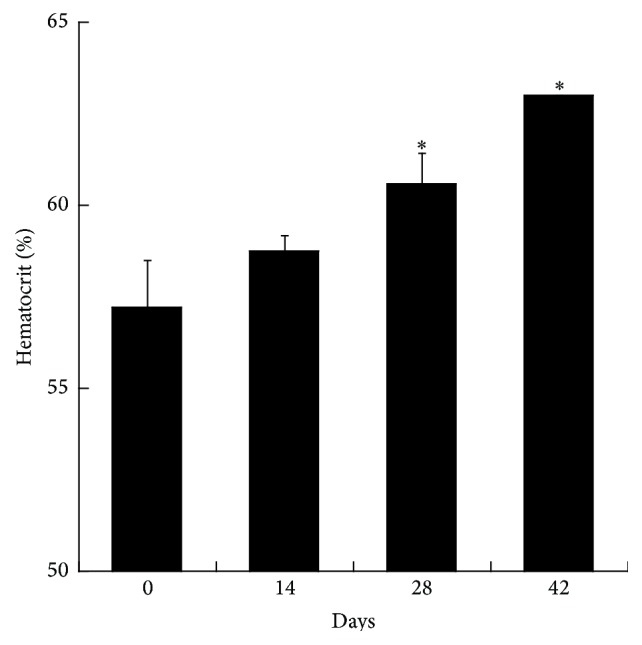
Hematocrit measured at four different time points of stored pRBCs. The bar chart demonstrates the increase in hematocrit measurements over the days of blood bag storage, including standard error bar (*p* value <0.05). The values shown are the mean ± SD of 3 independent experiments. ^*∗*^ *p* < 0.05, ANOVA compared to negative control cells.

**Figure 4:**
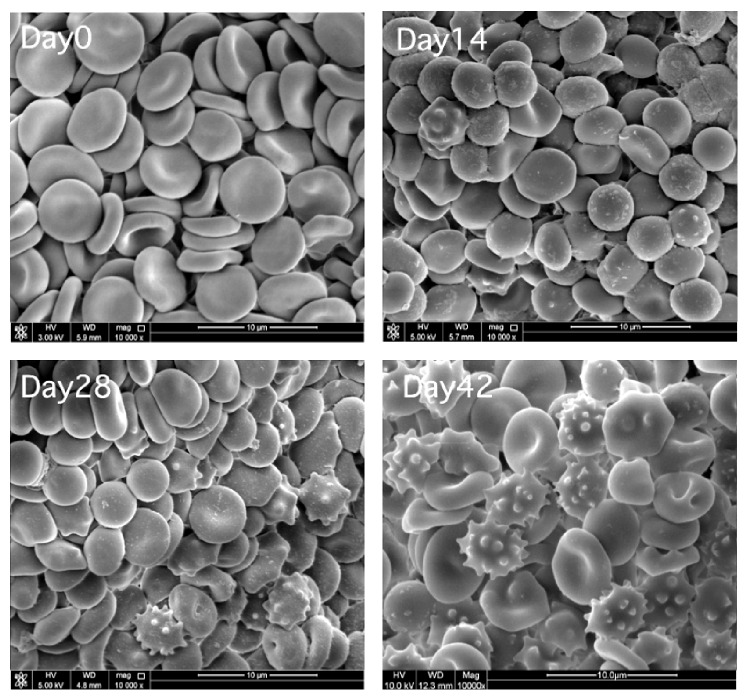
Scan electron micrograph of erythrocytes stored in blood bags with SAGM, 10000x magnification, representing changes of RBCs morphology in days 0, 14, 28, and 42. Scale bars and magnification values are reported in each panel.

**Figure 5:**
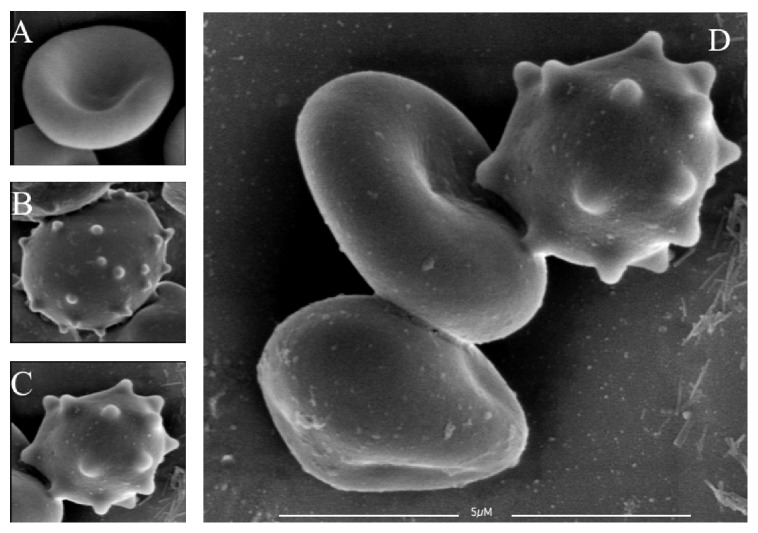
Scanning electron micrographs of erythrocytes showing gradual alteration into echinocytes. (A) Shows a normal erythrocyte on day 0. Cell (B) shows a primary echinocyte and (C) is a well-developed echinocyte. Micrograph (D) shows high magnification of progressive changes in RBC morphology on day 28. Echinocytes are morphologically altered red blood cells that appear to have abundant fine uniform spikes throughout the cell membrane.

The progressive increase in the hematocrit over the storage period was statistically significant (*p* < 0.05) which clearly illustrates an increase in hematocrit of the pRBC with SAGM in vitro, during the 42-day storage period. The hematocrit increased 6.6% in 42-day-old blood compared to fresh blood. The volume occupied by red blood cells in a certain volume of blood is referred to as the hematocrit and is commonly expressed as a percentage of the whole blood sample volume [9]. The hematocrit results in this study are shown in Figure 3. The hematocrit of day 0 was around 63% and increased to 70% at day 42. This increase can be explained by the morphological changes of the RBCs membrane, as can be seen in scanning electron microscope day 42 in Figures 4 and 5. The red blood cells lose parts of their membrane as microvesicles due to storage lesions, resulting in the decrease in the surface area-volume ratio and ending up with microcytic spherocytes. Besides, much of the RBCs populations are echinocytes with many membrane projections protruding outward, which prevents the normal packing of the RBCs. These abnormal red cells cause the plasma to be trapped in between the packing RBCs, accounting for 1–3% of the volume in microhematocrit method [10]. A study conducted by Antonelou et al. [4] was tested for hematocrit of stored leukoreduced blood bag on days 0, 20, 30, and 42. Their mean results showed an increase from 75.6% at day 0 to 77.0% at day 42. The hematocrit in this study does not reflect actual hematocrit of the donor on the day of blood donation; rather it is showing resistance of RBC packing in microhematocrit tube during centrifugation, due to extensive morphological alterations. In another study, it has shown that deformation in RBC morphology has effect on travelling human capillary like microchannels [11].

Scanning electron microscopy micrograph shows time points 0, 14, 28, and 42 of sample collection from leukoreduced packed RBCs bags, respectively (Figure 4). Day 0 is the control that shows normal morphology of erythrocytes with normal biconcave disc shape. As the storage time increases, changes can be noticed in day 14, and cells starts transforming to spherocytes and echinocytes. The number and severity of these cells increased in days 28 and 42. The extensive morphological alteration observed after 28 days appears to be irreversible.

### 3.3. Lipid Peroxidation

**Figure 6:**
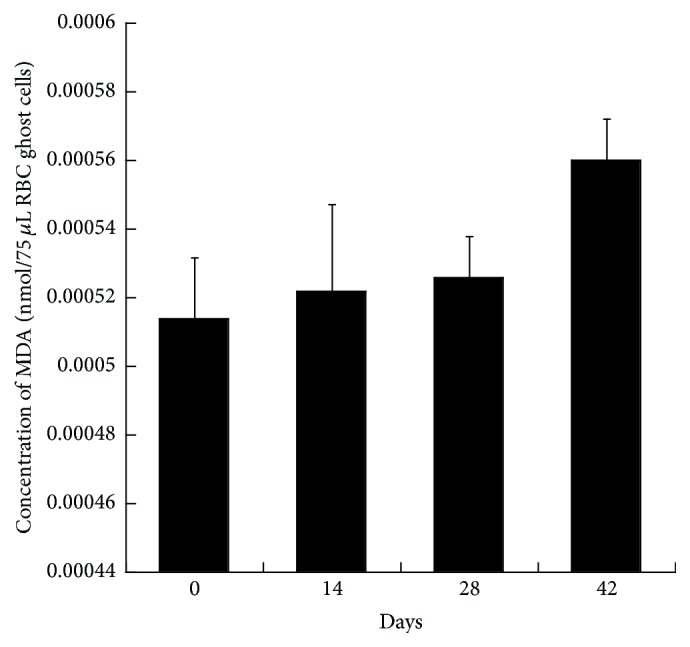
Demonstration of lipid peroxidation on stored pRBC. This data shows a slight increase of MDA concentration over the storage period. The values shown are the mean ± SD of 3 independent experiments. ^*∗*^ *p* < 0.05, ANOVA compared to negative control cells.

The concentration of malondialdehyde in the blood samples is demonstrated below.  Figure 6 shows a very slight increase of MDA in the blood stored over the period of 42 days. The standard error bars are overlapping and the *p* value >0.05, which concludes that the increase in the measurements is insignificant.

Malondialdehyde is one of the by-products resulting from lipid peroxidation of polyunsaturated fatty acids [12]. It is a thiobarbituric acid reactive substance that is measured by the TBARS assay. Measuring MDA is a useful marker in assessing the extent of lipid peroxidation [13]; thus, assessing the oxidative damage resulted by reactive oxygen species [14]. Oxidative damage occurs when the balance between antioxidants and prooxidants is lost, and, in the lipid peroxidation case, hydroxyl radicals resulting from Fenton reaction attack the fatty acids on the erythrocyte membrane ending up in weakening of the membrane's integrity [15, 16].

In this study, the results of TBARS assay conducted to assess the extent of lipid peroxidation of the RBCs membrane are illustrated in Figure 6. It shows that the MDA is increasing during the storage period. Although the increase is not significant, any increase indicates that lipid peroxidation occurred. Different protein expression patterns of the senescence markers due to oxidative stress in the RBCs seem to be accordingly related in stored blood [17].

A study about oxidative injury conducted by Chaudhary and Katharia [18] measured the MDA level on the blood samples over a storage time of 28 days. Their tests yielded results of mean MDA increased significantly from days 14 to 28, which indicated lipid peroxidation due to oxidative stress. Another study performed by Collard et al. [19] measured MDA concentration in pediatric packed RBCs bag at days 36 and 42 of storage [20]. The tests detected MDA presence in day 36 and that it showed a significant increase in day 42, which can be correlated with the occurrence of oxidative stress and lipid peroxidation that results in membrane damage. There are also studies which shows the role of antioxidants such as glutathione and glutathione peroxidase inhibits the oxidative damage to RBC due to protein or lipid oxidation [21]. In another study performed, integrated mass spectrometry based metabolomics and proteomics in stored red blood cells revealed considerable biochemical and structural alterations with continued storage time [22].

### 3.4. Clinical Impact of Transfused Old Blood

Frequent blood transfusion may expose high risk patient to adverse side effect as a result of receiving older blood. These adverse consequences can be associated with the metabolic, biochemical changes that red blood cells develop during storage. One of these side effects is related to RBCs losing their deformability during storage. In vivo, RBCs need to adapt their shape especially when passing through the capillaries and in the spleen in order to maintain their survival. During storage morphological alteration, biochemical changes and oxidative stress can damage the RBC membrane leading to impaired deformability. In addition, damage inflicted on the protein cytoskeleton can reduce the membrane elasticity increasing the RBCs fragility and reducing their viability [23]. Membrane loss by microvesiculation can reduce RBCs deformability in the circulation. Furthermore, these vesicles can contribute to posttransfusion effects by stimulate inflammatory response from the body and causing the blood to coagulate due to the externalized phosphatidylserine. The hemoglobin contained within these microvesicles can reduce nitric oxide (NO) availability in the recipient by binding to it leading to loss of vasoregulatory function of NO in the vessels. Reduced NO in the recipient can result in impaired endothelial function, platelet aggregation, and oxidative damage [24]. It has been also found that oxidized residues identified play a significant part in heme iron coordination, 2,3-DPG binding, and NO homeostasis [25]. Prolonged blood storage causes the accumulation of potassium in the supernatant which has been linked to the occurrence of cardiac arrhythmia in patients [26, 27]. This means that certain recipient such as pediatric patients or heart bypass surgery cannot be transfused without first removing the excess potassium [28]. Identifying the adverse effect associated with transfusing older blood is considered difficult because better understanding is required to correlate between storage lesions and the adverse effects in patients [29, 30].

## 4. Conclusion

Storage lesions and morphological alterations appeared to affect RBCs during the storage period. These lesions are caused by oxidative injury, biochemical alterations, and metabolic changes that result in irreversibly damaged and inflexible red blood cell membrane. Significant morphological alterations were observed during the 42 days of storage which was associated with the progressive increase in the osmotic fragility of the older blood. The morphological alteration was reflected by the increase of hematocrit with prolonged storage. Moreover, the increase in MDA level indicates that oxidative damage is occurring during storage which can further be linked to the morphological changes in the RBC. The changes that occur to RBCs during storage can be associated with posttransfusion effect on critically ill patients.
